# Spontaneous Eruption of Severely Impacted teeth: The Report of Two Cases

**DOI:** 10.4317/jced.55514

**Published:** 2019-05-01

**Authors:** Ildeu Andrade Jr., Marco-Aurélio-Benini Paschoal, Tamiris-de Oliveira Santos

**Affiliations:** 1 Associate Professor, Department of Orthodontics, Pontifical Catholic University of Minas Gerais, Belo Horizonte, MG, Brazil; 2 Adjunct Professor, Department of Pediatric Dentistry and Orthodontics, Federal University of Minas Gerais - UFMG, Belo Horizonte - MG, Brazil; 3 MS student, Graduate Program in Dentistry, Pontifical Catholic University of Minas Gerais, Belo Horizonte, MG, Brazil

## Abstract

The unerupted and impacted tooth is a common problem and the reason for many orthodontic and pediatric dental referrals, yet the approach to their management is still an area of controversy. This article presents two cases of severely impacted teeth that spontaneously erupted in the maxillary and mandibular arches. The first patient, a 9-year-old girl, presented a severe impaction of mandibular right and left second premolars. The second patient, a 7-year-old girl, presented with a severely impacted maxillary central incisor. In both cases, the teeth spontaneously erupted into excellent positions without surgical procedures and orthodontic traction. This raises important questions concerning the possible treatment options for such teeth as well as the timing of any interceptive treatment. In cases of unerupted or impacted teeth, a multidisciplinary approach is indicated involving orthodontics, paedodontics and oral surgery to establish the optimal treatment plan.

** Key words:**Tooth eruption, tooth, impacted, orthodontics, interceptive, case reports.

## Introduction

Tooth impaction is a relatively common clinical situation in dental offices and about 1% to 2% of orthodontic patients have impacted tooth (1). The permanent maxillary canines are the most frequently impacted teeth, followed by permanent third molars and mandibular second premolars (2). The lack of eruption of these elements may be associated with several factors, such as the presence of a supernumerary tooth, prolonged retention of the deciduous tooth, abnormal position of the impacted tooth, lack of space for eruption, root lacerations, alveolar or dental trauma and ankylosis (3-7). 

Several approaches have been purposed to treat these cases with different orthodontic appliances, mechanics and protocols (8,9), but little attention is given to the possible spontaneous irruption of the emerged teeth. It is generally known that there is a close relationship between the ability of a tooth to erupt and the level of dental root formation. A permanent tooth erupts from the alveolar bone when approximately two-thirds of the root have formed and then erupts into the oral cavity at approximately three fourths to complete root formation with a wide-open apex (10,11).

The spontaneous eruption of the impacted teeth may have an advantage over its surgical-orthodontic treatment approach. However, it is difficult to predict if spontaneous eruption will occur and how long it will take to emerge. The initial location and axial inclination of impacted teeth, the lack of space in the dental arch, the degree of root formation and the relation to adjacent teeth roots may influence the process of spontaneous eruption (12,13).

This article reports two clinical cases of unerupted permanent teeth based on minimally invasive approach.

## Case Reports

-Case 1

**Figure 1:**
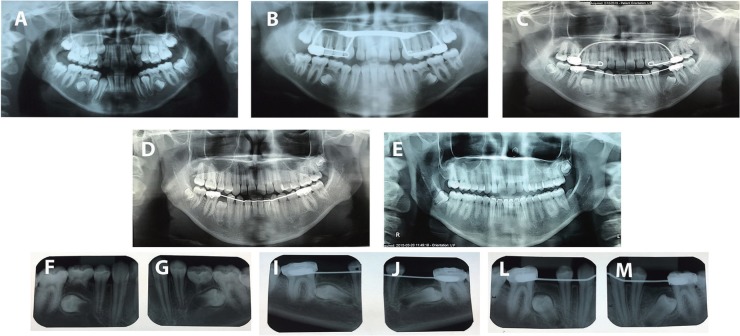
Panoramic and periapical radiographies monitoring of premolar eruption. A: Mandibular second premolars starting to turn backward. B: Mandibular second premolar 180º turned backward. C: Spontaneous uprighting, with mandibular lingual arch maintaining the E space. D: Eruption of mandibular second premolars. E: Final result with proper root parallelism. E-G: Mandibular second premolars 180º turned backward. I-J: Extraction of the mandibular primary second molars. L-M: Spontaneous uprighting of the mandibular second premolars.

A 9-year-old girl came to a private orthodontic office for an orthodontic evaluation with a chief complain of chewing impairment. After completion of medical and dental history, it was reported no systemic diseases or dental trauma history. In the intraoral examination, it was verified a skeletal Class I malocclusion, bilateral posterior crossbite, anterior open bite, and dental misalignment. The lateral cephalometric analysis showed skeletal Class I with vertical growth pattern and moderate maxillary retrusion and the panoramic radiograph showed the second premolars were still in the early stages of Nolla, with early signs of gyroversion (Fig. 1A).

Hence, correction of bilateral posterior crossbite and anterior open bite aiming to correct the eruption of the second lower premolars and to promote dental alignment was purposed.

The intervention was explained to the parents and was offered two treatment alternatives. The first option was to treat the patient in 2 phases, beginning with rapid maxilla expansion, vertical growth control with bite-block and chincup, and a lingual arch. The second phase included fixed orthodontic appliances for alignment and leveling, space closure, torque control, and root parallelism. The second option included treatment in a single phase, at the end of the mixed dentition with the same protocol above. Regarding the impaction of the mandibular second premolars, three options were considered: to wait for the spontaneous irruption, the surgical repositioning or the orthodontic traction of those teeth. The parents chose the first treatment option, which was a conservative approach for spontaneous irruption.

The treatment started with rapid palatal expansion with a bonded Hyrax. The objective was to correct the transverse maxillary deficiency and to control the vertical at the same time. Later on, a Hickham chin cup and elastics for maxillary protraction (300 g) was delivered to the patient, who was instructed to wear it 20 hs per day. The panoramic X-ray showed that the mandibular second premolars were turning backward in both sides (Fig. 1B). The follow-up x-Rays revealed that this condition was aggravating over time (Fig. 1 A-C; F-M). We decided to insert a lingual arch and refer the patient to her dentist for extraction of the deciduous mandibular second molar (Fig. 1C).

The first phase of the treatment was over and the lingual arch was kept in place until the eruption of the mandibular second premolars two years later (Fig. 1D). The comprehensive phase started after the fully eruption of the maxillary and mandibular second molars.

The post-treatment intraoral evaluation showed an Angle Class I occlusion with normal overjet, overbite, and canine and incisal guidance. The lower premolars spontaneously erupted without orthodontic traction. The posttreatment panoramic radiograph (Fig. 1E) shows that all spaces were closed without alveolar bone loss and root resorption. The case was retained by means of a maxillary wraparound retainer and a bonded premolar-to-premolar fixed retainer in the mandibular arch. The patient was instructed to only remove the retainer during periods of eating and tooth brushing. Treatment objectives were achieved with excellent esthetic and functional results. The patient became very happy with the results of her treatment, which was accomplished in 24 months.

-Case 2

**Figure 2:**
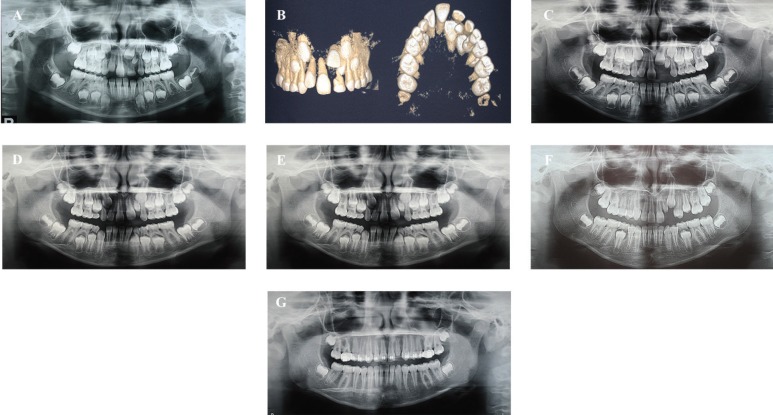
Panoramic radiographs and cone-beam computer tomography of the maxilla. A: Initial panoramic X-ray showing impaction of maxillary left central and lateral incisor. B: Initial cone-beam computer tomography showing the impaction of the maxillary left central and lateral incisor. C: Extraction of the primary maxillary left central incisor. D: Eruption of the permanent maxillary left central incisor. E: Extraction of the primary left canine and first molar, and eruption of the left first premolar. F-G: Maxillary left canine surpassing the eruption of the lateral incisor. Fixed appliances on the maxillary in order to manage the space for the left lateral incisor. Maxillary left canine erupted before the lateral incisor.

A 6-year-old girl, with the main complaint of unerupted incisors, was referred to a private dental clinic. Intraoral examination showed that the maxillary permanent left central and lateral incisors were missing. Furthermore, an Angle Class II malocclusion with anterior deep bite was verified with the patient presenting a convex profile and lower third of the face slightly decreased. The panoramic x-ray (Fig. 2A) and the cone-beam computer tomography (Fig. 2B) showed the impaction of the maxillary left central incisor and the impaction and shape abnormality of the maxillary left lateral incisor. Due to the clinical aspects, the aim of the treatment was to correct the Angle Class II malocclusion and anterior deep bite as well as to allow the eruption of the left central and lateral incisors.

The treatment plan involved the extraction of the maxillary left deciduous central incisor and two interceptive treatment alternatives were purposed: to keep the space for the development of the dentition and wait for the spontaneous irruption of the permanent teeth and the complete rhizogenesis of maxillary left canine. The second option was the orthodontic traction of these teeth. Her parents chose the most conservative approach, the first treatment option.

The patient was referred to the general dentist for extraction of the primary left maxillary central incisor (Fig. 2C). Despite the lack of the primary central incisor, the patient and her parents did not want to wear any retainer with a synthetic central incisor for esthetic reasons.

The permanent left maxillary central incisor erupted 12 months after the extraction of the primary incisors (Fig. 2D). At this time, the maxillary primary canines and first molars were extracted to allow the eruption of the permanent first premolars (Fig. 2E-F). Later on, the left canine erupted before the eruption the lateral incisors, and the rhizogenesis of the left lateral incisor was almost complete 36 months later (Fig. 2G-H). At that time, we decided to bond the fixed appliances on the maxillary arch in order to manage the space for the left lateral incisor. We are now waiting for the full eruption of this tooth to begin the comprehensive orthodontic treatment (Fig. 2H).

The Angle Class II malocclusion and anterior deep bite were totally corrected. At 1 year and 9 months of preservation, the permanent maxillary left central incisor totally irrupted. Yet, two years later, with almost complete rhizogenesis, the permanent maxillary left lateral incisor begun to erupt without the need for orthodontic traction (Fig. 2A-H).

## Discussion

Impaction of permanent teeth may be a challenge in the dental practice. The cases presented here demonstrated that sometimes less is more. Treatment options could include: invasive procedures, such as exteriorization of the tooth via surgical exposure or surgical repositioning with or without orthodontic traction; monitoring the eruption of the impacted teeth after the extraction of the primary teeth and finally, surgical removal of the unerupted tooth.. The choice of the appropriate treatment should consider a number of factors, including clinical and radiographic conditions: age and root formation stage, the presence of root dilaceration, position and sufficient space for the eruption. Moreover, it is necessary to evaluate the predecessor tooth, to check the presence of ankylosis and the possible root resorption degree of the deciduous teeth (14-16).

In order to choose an ideal treatment plan, different professionals are required, including pediatric dentists, orthodontists and oral surgeons, who are able to early identify the impaction of the tooth, to decide the ideal orthodontic mechanics, and to choose for the best surgical procedure, respectively.

In the first case, the mandibular second primary molars were ankylosed (Fig. 1). Keeping these teeth would not be a good option because ankylosed deciduous teeth tend to remain in infraocclusion which leads to the inclination of the adjacent teeth and possible impaction of the successor (7) Although there is an association between the deciduous retention and the degree of severity of the impaction of its successors (17), early extraction of deciduous can also cause complications such as lack of space. Due to the risk of loss of space after the early extraction of the ankylosed second primary molars, it was essential to preserve the “E” space with a space maintainer, which was done with a lingual arch. Failure to preserve this space could jeopardize the whole evolution of the case, highlighting the importance of interceptive treatments.

A previous study reported that spontaneous eruption will not occur if the tooth is 45 degrees away from the normal path of eruption (18,19). However, the mandibular second premolars of the first case presented a greater axial inclination and even so they erupted, which indicates that the degree of dental angulation is not a reliable parameter for evaluation of the probability of a spontaneous eruption.

The presence of impacted teeth is quite common. Regarding to the gender, both cases presented were related to female patients, which corroborates with the findings of Al-Abdallah *et al.* (17). These authors found that females suffer from more severe impaction of teeth, perhaps because their dental eruption commonly occurs a little earlier than in males.

The cases reported in this article involve growing patients, which allows the use of interceptive orthodontics. The optimal treatment time for Class II and Class III is still quite controversial, with no consensus on treatment in one or two phases. The two phases treatment protocol refers to an early phase with the use of functional orthopedic appliances and a second phase with fixed appliance treatment when all permanent teeth have erupted. The single-phase treatment, however, is only performed with fixed apparatus at a later age (20). To determine which protocol to follow, the orthodontist should evaluate important issues such as patient collaboration, level of complexity and time of treatment, the risk of dental fractures, financial and biological aspects, as well as psychological factors (21,22). This later, especially in cases of bullying, are important aspects to be considered not only in the choice of the orthodontic appliances for Class II and Class III treatment, but also to decide whether the orthodontic traction of the impacted tooth will be performed or if the spontaneous eruption can be expected, despite the long time that may be required. In our second case, the patient and her parents were informed about the long time required for the eruption of the permanent maxillary incisors and canine. They took the decision to wait and refused any esthetic protocol to camouflage the lack of those teeth in the smile.

Although impaction of maxillary permanent incisors is not as common as that of premolars2 it should receive special attention as they are important teeth for facial aesthetics. Zaid *et al.* identified spaced or missing teeth as the principal dentofacial features targets for bullying (23), which may have negative effects on the oral health-related quality of life (24,25).

Over the years, if nothing is done, there could be an increase in the severity of the impacted tooth (17). The satisfactory results obtained in the two cases here described, emphasize the importance of the early diagnosis and interceptive treatment, as well as the need for continuously clinical and radiographic follow-up. Moreover, combining the surgical procedure and orthodontic traction would require longer orthodontic treatment and may lead to trauma, especially in children, since surgical interventions are painful and require post-surgical care.

## Conclusions

The spontaneous eruption of impacted teeth may occur even in the severely impacted cases. Early diagnosis and interception at the right time are crucial to achieving successful results. The patient and parents must be aware of the long time required for the eruption of those teeth. Clinical and radiographic follow-ups are necessary during this time.
